# Association between internet use and private-sphere pro-environmental behavior among Chinese residents: an exploratory study from the perspective of ecological civilization construction

**DOI:** 10.3389/fpubh.2026.1852423

**Published:** 2026-06-03

**Authors:** Jie Gao, Xiaoqian Liang

**Affiliations:** 1 School of Marxism, Capital Normal University, Beijing, China; 2 School of Marxism, Tianjin University of Traditional Chinese Medicine, Tianjin, China

**Keywords:** ecological civilization construction, environmental risk perception, environmental self-efficacy, internet use, private-sphere pro-environmental behavior

## Abstract

**Introduction:**

This study uses data from the 2021 Chinese General Social Survey (CGSS) to examine the association between internet use and residents’ private-sphere pro-environmental behavior. Understanding the potential role of internet use in private-sphere pro-environmental behavior is crucial for designing effective environmental measures, protecting residents’ health, and advancing China’s ecological civilization construction in the digital age.

**Materials and methods:**

Using descriptive statistics and multiple linear regression analysis of a sample of 2,231 respondents, this study examined the association between internet use and private-sphere pro-environmental behavior. Further analyses were conducted to test the possible indirect associations of environmental self-efficacy and environmental risk perception between the two, while controlling for a series of sociodemographic variables.

**Results:**

Internet use was significantly and positively associated with private-sphere pro-environmental behavior, and robustness checks supported this finding. The indirect association of environmental risk perception between the two was significant, while no significant indirect association of environmental self-efficacy was found. However, environmental self-efficacy remained significantly and positively associated with private-sphere pro-environmental behavior.

**Conclusion:**

This study shows that environmental risk perception is an important variable for understanding the association between internet use and private-sphere pro-environmental behavior. At the same time, the findings suggest that internet use may not necessarily relate to environmental self-efficacy. Therefore, we recommend focusing on residents’ environmental risk perception, optimizing the content and format of environmental risk information online, and cultivating environmental self-efficacy through offline channels. These findings further deepen the understanding of the psychological mechanisms underlying the association between internet use and private-sphere pro-environmental behavior.

## 1 Introduction

The sustainable development of the ecological environment is closely linked to human health and well-being. Environmental pollution is a major factor affecting people’s lives and health. Research shows that air pollution is a key cause of cardiovascular and respiratory diseases (1, 2). In recent years, the Chinese government has placed greater emphasis on environmental governance, and overall environmental quality has continued to improve. However, environmental pollution remains in some areas (3). Under the 15th Five-Year Plan for National Economic and Social Development (2026–2030), promoting green development and achieving the dual carbon goals remain key tasks in advancing ecological civilization (4, 5).

The continued improvement of the ecological environment requires not only government governance but also the daily pro-environmental behaviors of residents. Private-sphere pro-environmental behavior refers to active actions taken by individuals or households to reduce environmental harm or negative impacts, such as green consumption, energy conservation, and waste disposal (6). Such behaviors play a positive role in reducing environmental pollution, lowering environmental health risks, and protecting individual health (7, 8). Existing research has built a rich empirical foundation for understanding the factors influencing pro-environmental behavior from psychological, situational, and sociodemographic perspectives. However, studies on the association between internet use and private-sphere pro-environmental behavior and its potential psychological mechanisms remain less systematic.

The internet has become deeply integrated into daily life. By 2025, China had over 1.1 billion internet users, with an internet penetration rate exceeding 80% (9). The internet is an important channel for information dissemination and has shown potential value in environmental health interventions. Research suggests that policy-driven digital health interventions are positively associated with health maintenance and environmental benefits, such as reduced carbon emissions and lower energy consumption (10). This suggests that the internet may serve as a potential medium for digital health promotion and may be linked to pro-environmental behavior through similar mechanisms. However, studies on the association between internet use and pro-environmental behavior have not yet reached a consistent conclusion (11–13). Given the complexity of the influence of internet use, it is necessary to carefully examine the association between the two and its underlying psychological mechanisms.

Among the possible psychological mechanisms, environmental self-efficacy and environmental risk perception serve as core variables. Both social cognitive theory and the theory of planned behavior emphasize that self-efficacy plays an important role in shaping individual behavior (14, 15). Studies have shown a positive association between environmental self-efficacy and pro-environmental behavior (16). Kasperson et al. (17) suggested that the media plays an important role in conveying risk information. Environmental risk perception is also positively associated with pro-environmental behavior (18). However, most existing studies have examined these two pathways separately, and research that incorporates them into a unified framework and compares their differential roles remains limited.

Based on the above analysis, this study focuses on examining the association between internet use and private-sphere pro-environmental behavior, while also exploring the possible roles that environmental self-efficacy and environmental risk perception may play between the two. The aim is to provide preliminary empirical evidence for reducing environmental health risks and promoting green lifestyles in the digital age.

This study makes two contributions. First, it incorporates environmental self-efficacy and environmental risk perception into the same analytical framework to examine whether they play a role in the relationship between internet use and private-sphere pro-environmental behavior, and to compare the differences between them. Second, starting from the specific context of China, it provides exploratory evidence for understanding the psychological mechanisms underlying individual pro-environmental behavior in the digital age, and offers preliminary empirical support for formulating policies related to residents’ health and environmental governance.

## 2 Literature background and conceptual framework

### 2.1 Internet use and pro-environmental behavior

Research has shown a positive association between internet use and individual pro-environmental behavior (11). Related studies have approached this issue from three main perspectives. First, online platforms offer advantages in providing information resources. A German study indicated that for obtaining correct information on the sorting of plastic food packaging, the internet and social media were consumers’ preferred information channels (19). Han et al. (20) found that user-generated content on social media containing environmental knowledge and awareness was positively associated with tourists’ environmental norms and their engagement in pro-environmental social media activities. Second, internet platforms play a role in social interaction. Han et al. (21) found that peer interaction on social media may promote individuals’ household waste sorting behavior. A Belgian study showed that for adolescents with higher injunctive peer norms, incidental exposure to climate content on social media was positively associated with their online climate engagement (22). Third, there are potential psychological pathways through which internet use is associated with pro-environmental behavior. Zhang and Gong (12) from the perspective of sustainable development, showed that internet use was positively associated with individuals’ environmental sustainability awareness, and this association was prominent in economically developed regions. A Singapore study found a positive association between internet attention and environmental citizenship participation (23).

According to social cognitive theory and the theory of planned behavior, information acquisition, social interaction, and cognitive factors jointly shape individual behavior. As an important platform that integrates these elements, the internet may be linked to pro-environmental behavior through multiple psychological pathways. Although existing research has confirmed the positive association between internet-based platforms and pro-environmental behavior from various perspectives, studies that take a holistic view and examine how internet use is associated with private-sphere pro-environmental behavior through different psychological pathways remain limited. Based on this, the study proposes the following hypothesis:

*Hypothesis 1*: Internet use is positively associated with private-sphere pro-environmental behavior among Chinese residents.

### 2.2 Internet use, environmental self-efficacy, and pro-environmental behavior

According to social cognitive theory, self-efficacy is a key factor in individual behavior change, influencing behavior through goal setting and effort (14). Existing studies suggest a complex relationship between internet use and environmental self-efficacy. On one hand, internet use is positively associated with environmental self-efficacy. Ison et al. (24) found that images of protective actions on social media had a positive effect on individuals’ sense of hope and self-efficacy. Wang et al. (25) showed that individuals’ prior information seeking on social media was positively correlated with both self-efficacy and environmental risk perception. On the other hand, information overload and negative content on the internet are negatively associated with environmental self-efficacy. Studies have shown that images of ecological threats on social media were negatively correlated with individuals’ self-efficacy (24). Han et al. (26) found that exposure to negative environmental information was negatively associated with environmental self-efficacy. In addition, some studies have not found a significant positive association between internet information exposure and environmental self-efficacy. Paek and Hove (27) found that media exposure was not directly associated with efficacy beliefs; rather, efficacy beliefs played a moderating role in the indirect path through which media exposure influenced pro-environmental behavioral intentions via risk perception or negative emotions.

Studies have shown that environmental self-efficacy is a core variable in explaining pro-environmental behavior. Wang (28) found that environmental efficacy was positively associated with individuals’ sustainable consumption behaviors. Wang and Hu (29) found that environmental efficacy was significantly and positively associated with residents’ low-carbon behaviors such as reducing car use and saving energy. One study showed that among rural older adults who had knowledge of climate change, their self-efficacy was positively associated with environmental actions aimed at adapting to and mitigating the effects of climate change (16). In summary, research that incorporates internet use, environmental self-efficacy, and private-sphere pro-environmental behavior into a unified framework to examine their associations remains relatively limited. Based on this, the study proposes the following hypothesis:

*Hypothesis 2*: Internet use is positively associated with environmental self-efficacy among Chinese residents.

*Hypothesis 2a*: Environmental self-efficacy is positively associated with private-sphere pro-environmental behavior among Chinese residents.

*Hypothesis 2b*: Environmental self-efficacy has an indirect association between internet use and private-sphere pro-environmental behavior among Chinese residents.

### 2.3 Internet use, environmental risk perception, and pro-environmental behavior

Environmental risk perception refers to the subjective judgments formed by the public when confronted with objective environmental risks (30). According to the social amplification of risk framework (SARF), risk signals may be amplified or attenuated through media, which in turn affects public environmental risk perception (17). Relevant studies have shown a positive association between internet use and environmental risk perception (31). Fan et al. (32) found that individuals who rely more on the internet for information may perceive higher levels of environmental pollution severity. One study showed that risk information on social media accompanied by visual data such as charts and tables could make individuals perceive environmental risks as greater (33).

Environmental risk perception plays an important role in pro-environmental behavior. Zhang et al. (34) found that when facing direct and immediate environmental risks at the individual level, people may be more likely to take pro-environmental actions. Another study showed that in areas frequently affected by heatwaves, heat risk perception was an important factor in rural residents’ pro-environmental behavior (35). Wang et al. (36) found that environmental risk perception was positively associated with individuals’ behavior to reduce plastic use. Notably, other studies have shown that high risk perception is not necessarily positively associated with individual pro-environmental behavior (37). Overall, however, studies that systematically examine the association between internet use and private-sphere pro-environmental behavior through environmental risk perception from a holistic perspective of internet use remain limited. Based on this, the study proposes the following hypothesis:

*Hypothesis 3*: Internet use is positively associated with environmental risk perception among Chinese residents.

*Hypothesis 3a*: Environmental risk perception is positively associated with private-sphere pro-environmental behavior among Chinese residents.

*Hypothesis 3b*: Environmental risk perception has an indirect association between internet use and private-sphere pro-environmental behavior among Chinese residents.

**Figure 1:**
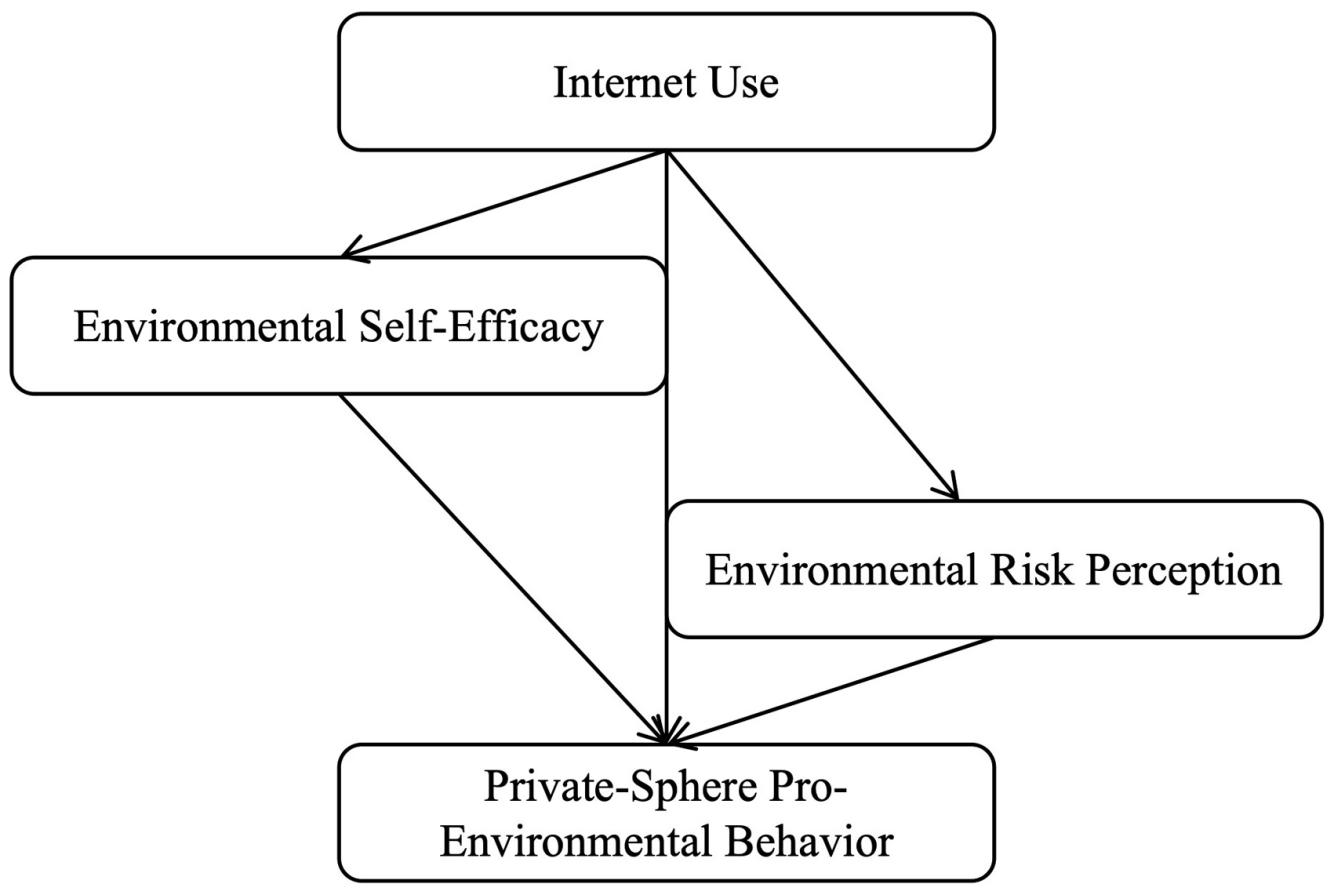
The conceptual framework of the research.

This study draws on social cognitive theory (SCT), the theory of planned behavior (TPB), and the value-belief-norm theory (VBN), and further adopts the social amplification of risk framework (SARF) as supplementary theoretical support. Through environmental self-efficacy and environmental risk perception, it systematically examines the association between internet use and private-sphere pro-environmental behavior (including waste recycling and green consumption) among Chinese residents. Figure 1 presents the theoretical framework.

## 3 Materials and methods

### 3.1 Data source

The data used in this study are derived from the Chinese General Social Survey (CGSS). The CGSS provides national, comprehensive, and continuous public datasets for research on social issues of theoretical and practical significance. This study adopts the 2021 CGSS data. The survey employed a multistage stratified sampling method and obtained a total of 8,148 valid responses. CGSS covers Chinese residents aged 18 and above. Given that the 2021 survey also included health modules from the East Asian Social Survey (EASS) and health and environment modules from the International Social Survey Program (ISSP), with each module randomly assigned to about one-third of the respondents, 5,407 cases that did not enter the modules were first removed, leaving 2,741 valid cases.

Based on this, we handled missing values for the core variables step by step. First, after treating missing values of the dependent variable (private-sphere pro-environmental behavior), where the absence of a recycling system in the residential area was treated as missing, we obtained 2,519 valid responses. Second, after removing “do not know” responses from the independent variable (internet use), we obtained 2,515 valid responses. Next, after further removing missing values of control variables (e.g., income), we obtained 2,231 valid responses. Finally, these were included as the final sample of 2,231 complete cases.

### 3.2 Variables selection

#### 3.2.1 Independent variable

The independent variable in this study is internet use. Following previous studies (11, 38), we measured residents’ internet use in terms of access. The measure was based on survey item A30b: “In the past 6 months, have you used the internet (including computers, smartphones, wearable devices, etc.)?” Responses were recoded as follows: “Have used the internet” was coded as 1, indicating “use the internet”; “Have not used the internet” was coded as 0, indicating “no use of the internet.” Other responses (e.g., “do not know”) were treated as invalid and excluded.

#### 3.2.2 Dependent variable

The dependent variable in this study is private-sphere pro-environmental behavior. Following previous studies (39, 40), we operationalized it with two specific items. These items cover two dimensions. The first is waste sorting behavior, measured by whether respondents purposely sort glass, aluminum cans, plastic, or newspapers for recycling (P19a). The second is green consumption behavior, measured by whether respondents purposely avoid buying certain products for environmental protection (P19b). Responses to both items were reverse coded as follows: “Always” = 3, “Often” = 2, “Sometimes” = 1, “Never” = 0. Respondents living in areas without recycling facilities were treated as missing because they could not perform waste sorting due to practical constraints. The scores of the two items were summed to create a composite index of private-sphere pro-environmental behavior.

#### 3.2.3 Mediating variables

This study uses two mediating variables: environmental self-efficacy and environmental risk perception. Following prior research (41, 42), environmental self-efficacy was measured with a single item from the survey: “People like me can hardly do anything to protect the environment” (P12-1). Because this item is reverse-worded, it was reverse-scored as follows: 5 for “strongly disagree,” 4 for “somewhat disagree,” 3 for “neither agree nor disagree,” 2 for “somewhat agree,” and 1 for “strongly agree.” A higher total score indicates a higher level of environmental self-efficacy among residents.

Environmental risk perception was measured using seven sources of pollution familiar to residents in their daily lives (P13a–g): vehicle exhaust, industrial emissions, pesticide and fertilizer use, river and lake pollution, climate change, genetically modified crops, and nuclear power plants. Each item was reverse-scored: 5 for “extremely harmful to the environment,” 4 for “very harmful,” 3 for “somewhat harmful,” 2 for “not very harmful,” and 1 for “not harmful at all.” Responses to the seven items were summed. A higher total score indicates a higher level of environmental risk perception among residents. Reliability analysis showed a Cronbach’s *α* of 0.773, indicating good internal consistency.

#### 3.2.4 Control variables

The control variables in this study include gender, age, political identity, education, income, marital status, and urban–rural status. Research suggests that these demographic variables may be directly or indirectly associated with pro-environmental behavior (43). Therefore, these seven variables were selected as controls. Descriptive statistics for each variable are shown in Table 1.

**Table 1:** Descriptive statistics for main variables (*N* = 2,231).

Variables	Observations	Mean	SD	Min	Max
Pro-environmental behavior	2,231	2.528	1.681	0.000	6.000
Internet use	2,231	0.706	0.456	0.000	1.000
Gender	2,231	0.474	0.499	0.000	1.000
Age	2,231	51.960	17.177	18.000	94.000
Political identity	2,231	0.137	0.344	0.000	1.000
Education	2,231	9.435	4.563	0.000	19.000
Income	2,231	8.222	4.167	0.000	16.118
Marital status	2,231	0.710	0.454	0.000	1.000
Urban–rural status	2,231	0.431	0.495	0.000	1.000
Environmental self-efficacy	2,231	3.190	1.181	1.000	5.000
Environmental risk perception	2,231	24.198	4.051	8.000	35.000

### 3.3 Methods

This study used Stata 17.0 to analyze the data and employed multiple linear regression models. Because the dependent variable (private-sphere pro-environmental behavior) and the mediating variables (environmental self-efficacy and environmental risk perception) were continuous, and the independent variable (internet use) was binary, ordinary least squares (OLS) regression was used for multiple regression analysis. To examine the association between internet use and private-sphere pro-environmental behavior, the baseline model, Equation 1 was specified as follows:


$${\mathrm{PEB}}_{i}=\beta_{0}+\beta_{1}{\mathrm{net}}_{i}+\sum \beta_{k}{\text{Controls}}_{\mathrm{ki}}+\varepsilon_{i}$$ (1)

In Equation 1, PEB*_i_* represents private-sphere pro-environmental behavior score, net*_i_* denotes internet use, controls*_ki_* stands for control variables including gender, age, political identity, education, income, marital status, and urban–rural status. *β*_0_ is the constant term, and *ε_i_* is the random error term. If *β*_1_ is significantly positive, it indicates that internet use is significantly and positively associated with residents’ private-sphere pro-environmental behavior.

Based on Equation 1, we sequentially added environmental self-efficacy, environmental risk perception, and both psychological variables to construct three additional models. The specifications for Equations 2–4 are as follows:


$${\mathrm{PEB}}_{i}=\beta_{0}+\beta_{1}{\mathrm{net}}_{i}+\sum \beta_{k}{\text{Controls}}_{\mathrm{ki}}+\beta_{9}\;{\mathrm{ESE}}_{i}+\varepsilon_{i}\;$$ (2)

$${\mathrm{PEB}}_{i}=\beta_{0}+\beta_{1}{\mathrm{net}}_{i}+\sum \beta_{k}{\text{Controls}}_{\mathrm{ki}}+\beta_{9}\;{\mathrm{ERP}}_{i}+\varepsilon_{i}\;$$ (3)

$$\begin{array}{l} {\mathrm{PEB}}_{i}=\beta_{0}+\beta_{1}{\mathrm{net}}_{i}+\sum \beta_{k}{\text{Controls}}_{\mathrm{ki}}\\ +\beta_{9}\;{\mathrm{ESE}}_{i}+\beta_{10}\;{\mathrm{ERP}}_{i}+\varepsilon_{i}\; \end{array}$$ (4)

The stepwise test and the bootstrap method were used to examine statistical relationships among the variables. First, we examined the correlation between internet use and private-sphere pro-environmental behavior. Second, we examined the correlations between internet use and the two psychological variables (environmental self-efficacy and environmental risk perception). Finally, we included both psychological variables in the model simultaneously to test whether the correlation between internet use and private-sphere pro-environmental behavior remained significant after controlling for these two variables, and whether the correlations between each of these two variables and private-sphere pro-environmental behavior were significant. On this basis, the bootstrap method was further used to assess the indirect associations between internet use and private-sphere pro-environmental behavior through the two mediating variables.

## 4 Results

### 4.1 Statistical analysis

#### 4.1.1 Baseline regression analysis

Model 1 examined the overall association between internet use and private-sphere pro-environmental behavior. The regression results showed a significant positive association between the two (*β* = 0.240, *p* < 0.05). After controlling for other variables, internet users scored 0.24 points higher on pro-environmental behavior than non-users. Thus, Hypothesis H1 was supported.

Model 2 added environmental self-efficacy to Model 1. The results showed that internet use remained significantly and positively associated with private-sphere pro-environmental behavior (*β* = 0.244, *p* < 0.05), and environmental self-efficacy was significantly and positively associated with private-sphere pro-environmental behavior (*β* = 0.238, *p* < 0.001). These results tentatively suggest that environmental self-efficacy may have an indirect association between the two, which requires further testing in subsequent models.

Model 3 added environmental risk perception to Model 1. The results showed a positive association between internet use and private-sphere pro-environmental behavior (*β* = 0.188, *p* = 0.061), and environmental risk perception was significantly and positively associated with private-sphere pro-environmental behavior (*β* = 0.048, *p* < 0.001). These results tentatively suggest that environmental risk perception may have an indirect association between the two, which requires further testing in subsequent models.

Model 4 included both environmental self-efficacy and environmental risk perception simultaneously, forming a full parallel two-mediator model. The results showed that internet use remained significantly and positively associated with private-sphere pro-environmental behavior (*β* = 0.198, *p* < 0.05), and both environmental self-efficacy (*β* = 0.225, *p* < 0.001) and environmental risk perception (*β* = 0.043, *p* < 0.001) were significantly and positively associated with private-sphere pro-environmental behavior. Whether the indirect associations hold requires further examination of the associations between the independent variable and the mediators, as well as a bootstrap test of the statistical significance of the indirect associations (see Table 2).

**Table 2:** Baseline regression analysis for main variables.

Variables	Model 1	Model 2	Model 3	Model 4
Internet use	0.240** (0.101)	0.244** (0.099)	0.188* (0.100)	0.198** (0.099)
Environmental self-efficacy		0.238*** (0.031)		0.225*** (0.031)
Environmental risk perception			0.048*** (0.009)	0.043*** (0.009)
Gender	−0.064 (0.073)	−0.046 (0.072)	−0.034 (0.073)	−0.021 (0.072)
Age	0.005* (0.003)	0.007** (0.003)	0.004 (0.003)	0.007** (0.003)
Political identity	0.120 (0.111)	0.012 (0.110)	0.105 (0.110)	0.003 (0.110)
Education	0.030*** (0.011)	0.020* (0.011)	0.024** (0.011)	0.015 (0.011)
Income	0.014 (0.009)	0.015* (0.009)	0.013 (0.009)	0.014 (0.009)
Marital status	0.186** (0.080)	0.164** (0.079)	0.191** (0.079)	0.171** (0.078)
Urban–rural status	0.293*** (0.083)	0.294*** (0.082)	0.265*** (0.082)	0.269*** (0.081)

Control variables	Control	Control	Control	Control
*N*	2,231	2,231	2,231	2,231
Adj. *R*^2^	0.032	0.057	0.044	0.067
*F*	10.296	16.103	12.512	16.963

*N* = 2,231; Coefficients are unstandardized regression coefficients; numbers in parentheses are standard errors; *p < 0.1; **p < 0.05; ***p < 0.01.

### 4.2 Mediation analysis

To further examine the association between internet use and private-sphere pro-environmental behavior, this study used a stepwise testing approach combined with the bootstrap method (95% confidence interval) to test the indirect association between the two. Tables 3, 4 present the corresponding results.

**Table 3:** The indirect association of environmental self-efficacy between internet use and private-sphere pro-environmental behavior.

Variables	Pro-environmental behavior	Environmental self-efficacy	Pro-environmental behavior
Internet use	0.240** (0.101)	−0.016 (0.069)	0.244** (0.099)
Environmental self-efficacy			0.238*** (0.031)

Control variables	Control	Control	Control
*N*	2,231	2,231	2,231
Adj. *R*^2^	0.032	0.084	0.057
*F*	10.296	26.415	16.103

*N* = 2,231; Coefficients are unstandardized regression coefficients; numbers in parentheses are standard errors; *p < 0.1; **p < 0.05; ***p < 0.01.

**Table 4:** The indirect association of environmental risk perception between internet use and private-sphere pro-environmental behavior.

Variables	Model 1: Pro-environmental behavior	Model 2: Environmental risk perception	Model 3: Pro-environmental behavior
Internet use	0.240** (0.101)	1.071*** (0.238)	0.188* (0.100)
Environmental risk perception			0.048*** (0.009)

Control variables	Control	Control	Control
*N*	2,231	2,231	2,231
Adj. *R*^2^	0.032	0.069	0.044
*F*	10.296	21.618	12.512

*N* = 2,231; Coefficients are unstandardized regression coefficients; numbers in parentheses are standard errors; *p < 0.1; **p < 0.05; ***p < 0.01.

Table 3 shows that the regression coefficient from internet use to environmental self-efficacy was −0.016 (*p* = 0.819), indicating no significant statistical association between the two. The bootstrap test showed an indirect association coefficient of −0.004 (95% CI [−0.036, 0.029]), with the confidence interval containing zero. Therefore, the indirect association of environmental self-efficacy was not statistically significant. Hypotheses H2 and H2b were not supported. However, environmental self-efficacy was significantly and positively associated with private-sphere pro-environmental behavior (*β* = 0.238, *p* < 0.001), supporting Hypothesis H2a.

Table 4 shows that the regression coefficient from internet use to environmental risk perception was 1.071 (*p* < 0.001), and the regression coefficient from environmental risk perception to private-sphere pro-environmental behavior was 0.048 (*p* < 0.001). The bootstrap test showed an indirect association coefficient of 0.052 (95% CI [0.022, 0.081]), not containing zero, and a direct association coefficient of 0.188 (95% CI [−0.009, 0.386]), containing zero (*p* = 0.062). The indirect association of environmental risk perception between internet use and private-sphere pro-environmental behavior was statistically significant. Hypotheses H3, H3a, and H3b were all supported.

### 4.3 Robustness test

The robustness checks in this study were conducted using the following three methods. Results are shown in Table 5.

**Table 5:** Robustness test for main variables.

Variables	Model 1	Model 2	Model 3	Model 4	Model 5	Model 6	Model 7	Model 8
IU	0.274** (0.109)	0.220** (0.110)	0.222** (0.099)	0.181* (0.098)	0.202*** (0.076)	0.170** (0.075)	0.195*** (0.055)	0.166*** (0.054)
ESE		0.258*** (0.034)		0.217*** (0.030)		0.178*** (0.023)		0.123*** (0.017)
ERP		0.049*** (0.010)		0.044*** (0.009)		0.033*** (0.007)		0.029*** (0.005)

Control variables	Control	Control	Control	Control	Control	Control	Control	Control
*N*	2,231	2,231	2,231	2,231	2,231	2,231	2,231	2,231
Adj. *R*^2^			0.061	0.094	0.042	0.078	0.038	0.078
*F*			14.081	18.789	13.178	19.931	11.988	19.954

*N* = 2,231; IU, internet use; ESE, environmental self-efficacy; ERP, environmental risk perception. Unstandardized coefficients are shown; standard errors in parentheses. *p < 0.1; **p < 0.05; ***p < 0.01.

First, we changed the regression model (see Models 1 and 2). We re-estimated the model using an ordered logistic model. The results showed that internet use was significantly and positively associated with pro-environmental behavior (*β* = 0.274, *p* < 0.05). After adding the mediating variables, the coefficient decreased to 0.220 (*p* < 0.05), and both mediators remained significantly positive (*p* < 0.001). This indicates that the main conclusions are robust to different model specifications.

Second, we reduced omitted variable bias (see Models 3 and 4). We added region dummies (using the eastern region as the reference group, and controlling for central, western, and northeastern regions). After controlling for region fixed effects, internet use remained significantly and positively associated with the outcome (*β* = 0.222, *p* < 0.05). When the mediators were added, its coefficient decreased, but both mediators were still significantly positive (*p* < 0.001). This suggests that the main conclusions are not affected by controlling for regional differences.

Third, we changed the operationalization of the dependent variable (Models 5–8). Two alternative measures were used: the pro-environmental behavior score recoded into a 0–4 scale (Models 5-6); and willingness to sort waste as the dependent variable (1–5 scale, Models 7-8, as a supplementary analysis). Under both measures, the positive association of internet use was significant (*β* = 0.202, *p* < 0.01; *β* = 0.195, *p* < 0.001). After adding the mediators, the coefficients of internet use decreased to 0.170 (*p* < 0.05) and 0.166 (*p* < 0.01), respectively, and both mediators remained significantly positive (*p* < 0.001). This shows that the main conclusions do not depend on the specific operationalization of the dependent variable.

Taken together, the results of these robustness checks are consistent with the main analysis, indicating that the core conclusions are robust.

## 5 Discussion

This study uses data from the China General Social Survey 2021. After controlling for sociodemographic variables, it empirically examines the association between internet use and private-sphere pro-environmental behavior. It then discusses the findings from both theoretical and practical dimensions within the context of China’s ecological civilization construction.

### 5.1 Theoretical dimension

The study found a significant positive association between internet use and private-sphere pro-environmental behavior (*β* = 0.240, *p* < 0.05). This result may be related to the fact that individuals who use the internet have stronger environmental awareness (12). It may also be attributed to the ease with which people can access, exchange, and discuss environmental information online (44). Furthermore, in the context of China’s ongoing ecological civilization construction, residents’ demand for a higher quality of life and health protection may lead them to pay more attention to environmental information on the internet, thereby making them more likely to adopt pro-environmental behaviors (45). These findings are consistent with Gong et al.’s (11) findings.

There was no positive association between internet use and environmental self-efficacy (*β* = −0.016, *p* = 0.819), and no indirect association of environmental self-efficacy between internet use and private-sphere pro-environmental behavior was found. This echoes Paek and Hove’s (27) findings, where efficacy beliefs played mainly a moderating rather than a mediating role in the model. The relationship between internet use and environmental self-efficacy is complex. On the one hand, internet use may be positively or negatively associated with environmental self-efficacy (negative information online may weaken environmental self-efficacy) (24). On the other hand, self-efficacy may also influence internet use in reverse (46, 47), meaning that there is not necessarily a positive association between the two. Notably, although no positive association was found between internet use and environmental self-efficacy, environmental self-efficacy remained positively associated with private-sphere pro-environmental behavior (*β* = 0.238, *p* < 0.001). This result is consistent with Sorour et al.’s (16) study. The finding suggests that environmental self-efficacy is an important variable for private-sphere pro-environmental behavior.

The indirect association of environmental risk perception between internet use and private-sphere pro-environmental behavior was statistically significant. The results showed a positive association between internet use and environmental risk perception (*β* = 1.071, *p* < 0.001). This may be because environmental knowledge, reports of risk events, and environmental issues disseminated on internet platforms are more likely to arouse residents’ risk awareness (32). There was also a positive association between environmental risk perception and pro-environmental behavior (*β* = 0.048, *p* < 0.001), meaning that residents with a higher level of environmental risk perception may be more inclined to take pro-environmental actions. This finding is consistent with previous research (36). Taken together, these results suggest that internet users may have a higher level of environmental risk perception and may be more likely to engage in pro-environmental behaviors, which aligns with Zhou and Tang’s (31) findings.

### 5.2 Practical dimension

To optimize the content and format of environmental risk information dissemination, it is recommended to fully recognize the positive significance of individual environmental risk perception. Relevant authorities can use internet platforms to disseminate local environmental information—such as air quality indexes, extreme weather warnings, and related health impacts—in a simple and visual format. At the same time, social media can be used to deliver tailored risk content. This may help raise residents’ environmental risk awareness and make them more likely to take pro-environmental actions (e.g., sorting waste voluntarily). Priority should be given to authoritative data sources, and platforms should be encouraged to implement tiered management of environmental content and label it with credibility tags. Moreover, online communication should avoid over-dramatizing risks. Instead, risk information should be followed by corresponding solutions, helping residents acquire the knowledge and skills needed to cope with environmental risks.

Focus on residents’ environmental mental health and cultivate their environmental self-efficacy in a targeted way. Although this study found no statistically significant association between internet use and environmental self-efficacy, self-efficacy was positively associated with private-sphere pro-environmental behavior. In the context of the digital age, it is advisable to appropriately combine online and offline methods and integrate the cultivation of self-efficacy into ecological civilization education programs. This can be done by providing targeted offline training and engaging ecological education activities, as well as by integrating ecological concepts from fine traditional Chinese culture, such as “harmony between nature and humans,” into exemplary cases of individual pro-environmental actions and promoting these cases to enhance residents’ confidence in their ability to act pro-environmentally.

## 6 Limitations

Although this study tentatively finds that internet use may have positive implications for residents’ private-sphere pro-environmental behavior, the following limitations remain. First, this study used cross-sectional data from the CGSS 2021, which cannot establish causal directions among the variables. Future research could use longitudinal or experimental designs to test causal relationships. Second, there are limitations in the measurement and selection of key variables. Internet use was measured as a binary variable based on whether respondents had access to the internet. This operationalization does not capture intensity information such as usage frequency or duration, and therefore cannot test dose–response relationships (e.g., whether more frequent use is associated with higher levels of pro-environmental behavior). Due to data constraints, the dependent variable included only two items, which does not cover other aspects of private-sphere pro-environmental behavior (e.g., energy saving, water conservation). The measure of environmental self-efficacy was based on a single item, and the results may be influenced by the measurement instrument. Future research should adopt more refined measures of internet use (e.g., weekly usage time, specific types of applications) and use broader, validated scales of pro-environmental behavior. In addition, environmental self-efficacy could be measured with a more established multi-item scale, distinguishing between internal efficacy (knowledge, skills) and external efficacy (concern about infrastructure and policies), so as to more comprehensively examine the association between internet use and environmental self-efficacy. Third, omitted variables may have influenced the estimates. For example, confounders such as environmental concern, environmental knowledge, and community infrastructure were not controlled for. Future research could include these variables. In summary, because the data are cross-sectional, the results should be viewed as exploratory evidence, and caution is needed when generalizing the conclusions.

## 7 Conclusion

Based on the background of China’s ecological civilization construction and using data from the CGSS 2021, this study found a positive association between internet use and residents’ private-sphere pro-environmental behavior. Environmental risk perception had a significant indirect association between the two. This finding suggests that environmental risk perception may be an important factor in the relationship between internet use and private-sphere pro-environmental behavior. In addition, although no indirect association of environmental self-efficacy was found between internet use and private-sphere pro-environmental behavior, environmental self-efficacy still showed a positive association with private-sphere pro-environmental behavior. These results suggest that there may not necessarily be a direct link between internet use and environmental self-efficacy. This study provides preliminary empirical evidence for understanding the psychological mechanisms underlying residents’ pro-environmental behavior in the digital age, and offers insights for discussions on environmental governance, health maintenance, and the promotion of green lifestyles.

## Data Availability

The original contributions presented in the study are included in the article/supplementary material, further inquiries can be directed to the corresponding author.
